# Investigation on the Crystallization Behaviors of Polyoxymethylene with a Small Amount of Ionic Liquid

**DOI:** 10.3390/nano9020206

**Published:** 2019-02-05

**Authors:** Qi Jiao, Qin Chen, Lian Wang, Hualin Chen, Yongjin Li

**Affiliations:** 1 Chengdu Institute of Organic Chemistry, Chinese Academy of Sciences, Chengdu 610041, China; jiaoqisncc@sina.com (Q.J.); aofly@163.com (H.C.); 2 University of Chinese Academy of Sciences, Beijing 100049, China; 3 Coal Chemical Industry Technology Research Institute, Shenhua Ningxia Coal Group Co. Ltd., Yinchuan 750411, China; 4 College of Materials, Chemistry and Chemical Engineering, Hangzhou Normal University, No. 2318 Yuhangtang Rd., Hangzhou 311121, China; 15757116907@163.com (Q.C.); yongjin-li@hznu.edu.cn (Y.L.)

**Keywords:** polyoxymethylene, ionic liquid, crystallization behavior, nucleation

## Abstract

Polyoxymethylene (POM) blends with excellent stiffness–toughness balance are successfully developed using Tributyl(octyl)phosphonium bis(trifloromethanesulfonyl) imide (TBOP-TFSI), one type of room-temperature ionic liquid, as the nucleating agent. Crystallization behaviors of POM blends have been studied by differential scanning calorimetry (DSC) and polarized light microscopy (PLM). The incorporation of TBOP-TFSI induces the crystal nucleation and fine crystal grain of POM, and also a much shorter hemi-crystalline time with only 0.5 wt% addition. The nucleation effect of ionic liquid leads to considerable improvement in the impact strength of POM blends while not sacrificing its tensile strength. Moreover, antistatic properties with a long-time stable performance are achieved by TBOP-TFSI addition as the electrical resistance reaches 10^11^ Ω/sq.

## 1. Introduction

Polyoxymethylene (POM) is one of the most important engineering plastics and is broadly used in many areas, such as automobiles, the coal industry, and also in architecture materials [1,2,3,4,5,6]. POM is a kind of weakly polar linear crystalline polymer, and its molecular structure is: [CH_2_O]_n_. Due to its highly regularly structured molecular chain, POM can easily crystallize to form spherulites, and exhibits high crystallinity. The main reason for its excellent performance is that POM has the high density and crystallinity. For example, POM has a high strength and flexural modulus, excellent chemical and creep resistance, and excellent dimensional stability, wear and friction properties. Though high crystallinity of POM brings excellent mechanical properties, the large spherulites of POM also lead to low impact toughness and gap-sensitivity. Moreover, POM has a high surface resistivity of about 10^14^ to 10^17^ Ω/sq. Such high resistivity leads to easily accumulated electrostatic charges on the surface of POM. Such features limit the application of POM in industry and daily life [4,5,6,7,8,9,10]. Improving its crystallization performance can enhance the properties of POM and expand the application in industry. 

Many scientists have investigated the effects of nucleating agents on the crystallization performance of POM [6,11,12,13,14,15,16,17,18,19]. Zhao [19] showed that multi-walled carbon nanotubes (MWCNTs) had an excellent nucleation effect on POM, which lead to an increase in crystallization temperature and crystallization rate. Hu [20] showed the isothermal and non-isothermal crystallization behavior and morphology of POM blended with a small amount of polyamide (PA) as the nucleating agent. They illustrated that the addition of PA reduced the spherulite size and improved the crystallization growth rate and the crystallinity of POM due to the nucleation effect of PA on POM. 

Recently, ionic liquids, have been proposed as lubricants [21], plasticizers [22], and surfactants [23] in many polymer blends as a new type of modification agent due to their low melting point, low toxicity, and high chemical and physical stability. Ionic liquids have also been reported as nucleating agents for a few semicrystalline polymers. Liu [24] reported one pyrrolidinium ionic liquid served as the nucleating agents for PET and shortened induction time and enhanced crystallization rate. Previously, it is reported by our group that a kind of ILs, 1-butyl-3-methylimidazolium hexafluorophosphate [BMIM][PF6], can increase the γ-phase content of poly(vinylidene fluoride) (PVDF) by simply mix blending, which indicates [BMIM][PF6] played a role as a γ-phase nucleating agent in these blends [25]. However, literatures on the nucleation effect of ionic liquids on crystallization behavior for other semicrystalline polymers are quite limited. 

Moreover, ionic liquids have been utilized as antistatic agents due to their excellent ionic conductivity. Combining conductive agents into insulative polymers is of some importance to prevent electrostatic-charge accumulation on the surface of neat polymers, especially for those applied in electronic devices [26,27]. Pernak [28] et al. first incorporated ionic liquid into polyethylene matrix to investigate antistatic ability related to the chemical structure of ionic liquid. Since then, ionic liquids have been reported as antistatic agents for various polymers, such as polypropylene [12,29], poly(vinylidene flouride) [25,30], polystyrene [31], and also biodegradable poly(_L_-lactic acid) (PLLA) [32]. It is believed compatibility and interaction between ionic liquid and matrix are of crucial importance when designing antistatic composites. Ionic liquids with moderate compatibility or intermolecular interactions should be considered as suitable modification agents.

In this work, we used Tributyl(octyl)phosphonium bis(trifloro methanesulfonyl) imide (TBOP-TFSI, short for convenience as TBOP-TFSI), one type of room-temperature ionic liquid (RTIL), as a nucleating agent and antistatic agent, which showed a partial compatibility and a weak ion–molecular interaction with the matrix to obtain multi-functional POM materials by simple mixing. It was found that small amounts of TBOP-TFSI could facilitate the nucleation of POM, and decrease the sizes of POM spherulites, which resulted in the improvement in the toughness of POM. More importantly, the tensile strength of composites did not decrease. Moreover, the POM composite achieved antistatic effects by adding only 0.5 wt% TBOP-TFSI. In a word, we fabricated antistatic POM composites with simultaneously excellent stiffness–toughness balance.

## 2. Materials and Methods

### 2.1. Materials

POM (MC 90) used was commercially available from Shenhua Co., Ltd. (Yinchuan China). TBOP-TFSI was supplied by Solvay Co., Ltd (Brussels, Belgium). All of the samples were used as received.

### 2.2. Preparation of POM/TBOP-TFSI Blends

All materials were vacuum-dried at 80 °C for 12 h before use. The composites were prepared by extruder with single screw at 170 to 200 °C with a rotation speed of 50 rpm after simply mixing physically. After Extrusion, some samples were hot-pressed at 200 °C under a 10 MPa pressure for 3 min to a 300 μm film, followed by cool-pressing for 2 min at room temperature. The obtained sheets were used for the following characterization. The specimens for tensile tests and notched impact test were prepared by injection molding. The IL loadings in this work were calculated based on only the amount of matrix POM by weight.

### 2.3. Characterization

#### 2.3.1. Antistatic Properties

Electrical conductivity was tested by an ultrahigh resistivity meter, using a piece of URS probe electrode (MCPHT450) at 100 V with a 300 μm-thickness sample.

#### 2.3.2. Mechanical Properties

Tensile test were measured by Instron universal material testing system (model 5966) at 25 °C. The tensile rate was 5 mm/min. The notched impact test was performed according to the GB/T16420-1996 standard. Samples for tensile test and notched impact test were prepared by injection molding (Haake MINIJET PRO).

#### 2.3.3. Scanning Electron Microscopy

The microstructure of cross-fractured surface of samples was obtained using field emission scanning electron microscopy (FESEM, SEM-JSM 6700). The acceleration voltage was 3 kV and the fractured surface was coated with a thin layer of gold before the SEM observation. 

#### 2.3.4. Fourier Transfrom Infrared (FTIR)

The Fourier transfrom infrared (FTIR) measurements were carried out in transmittance mode on grinding samples by FTIR spectroscopy (FTIR, Bruker Tensor). The FTIR spectra were recorded at a resolution of 2 cm^−1^, and 64 scans from 4000 to 400 cm^−1^ were averaged. The samples were prepared by spin-coating after dissolving neat POM and the blends in hexafluoroisopropanol.

#### 2.3.5. Differential Scanning Calorimetry

The differential scanning calorimetry (DSC) measurements were carried out by a differential scanning calorimetry (DSC, TA-Q2000). Some samples were first heated to 200 °C and held for 5 min to eliminate previous thermal history, then cooled to 20 °C. Both the cooling and heating rates were 10 °C/min. The others were first heated to 200 °C like the previous one, then cooled to 152 °C as fast as possible for isothermal crystallization process. The experiments were carried out under a continuous high purity nitrogen atmosphere. 

#### 2.3.6. Polarized Light Microscopy

The morphologies of the POM spherulites were observed by polarizing light microscopy (PLM, Olympus BX51) equipped with a digital camera, using a Linkham LTS 350 hot stage to control the temperature at 152 °C. All samples were spin-coated onto clean glass sheets, and the thickness of samples on the glass sheet was about 10 mm.

## 3. Results

### 3.1. Morphologies of POM/TBOP-TFSI Blends and the Interactions between POM and TBOP-TFSI

**Figure 1:**
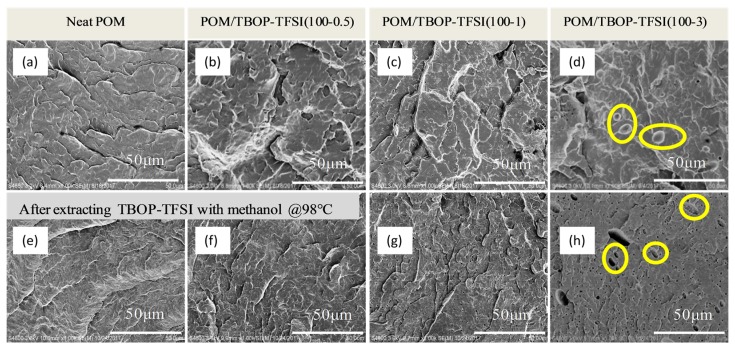
SEM images of fracture surface morphologies of polyoxymethylene (POM)/tributyl(octyl)phosphonium bis(trifloromethanesulfonyl) imide (TBOP-TFSI) blends with indicated TBOP-TFSI loadings before (**a**–**d**) and after extracting with methanol (**e**–**h**).

Figure 1 shows SEM images of fracture surface morphologies of POM/TBOP-TFSI thin films with indicated TBOP-TFSI loadings. No visible phase was observed for the blend containing 0.5 wt% ionic liquid before or after methanol extraction, suggesting a homogeneous distribution of TBOP-TFSI within POM matrix. However, several domains of less than 100 nanometers were observed for blending with 1 wt% ionic liquid loading, indicating a phase-separated structure. Increasing TBOP-TFSI loading induced the aggregation of ionic liquid and domain formation in the matrix. A typical sea-island structure was observed at 3 wt% TBOP-TFSI loading, of which domain size of ionic liquid became over several hundred nanometers. Some anomalous domains could be seen for this sample after extracting ionic liquid by methanol at 98 °C for 24 h, implying the partial compatibility nature of POM and TBOP-TFSI. It is believed that the dispersion of TBOP-TFSI within matrix was responsible for several properties as surface conductivity of binary blends. POM and TBOP-TFSI remained compatible at very low ionic liquid content. Consequently, an electrical conducting pathway could be formed due to the high mobility of ionic liquid. However, for blends containing more than 1 wt% of ionic liquid, typical sea-island structures were formed due to phase separation between ionic liquid and matrix. 

**Figure 2:**
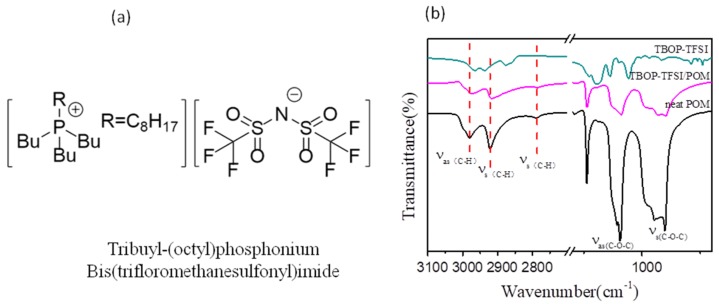
(**a**) Molecular structure of TBOP-TFSI; (**b**) Fourier transfrom infrared (FTIR) spectra of POM with TBOP-TFSI.

We then utilized FTIR to observe the intermolecular interaction between POM and TBOP-TFSI. Figure 2a shows the molecular structure of TBOP-TFSI, Figure 2b shows the FTIR spectra of POM/TBOP thin films. It is found that the absorption bands appearing at 2922 cm^−1^ and 2790 cm^−1^ are due to the symmetric stretching vibration of C-H, and the absorption peak at 2979 cm^−1^ is assigned to the C-H asymmetric stretching vibration of POM [33]. The three peaks shift to lower wavenumbers for POM/TBOP-TFSI mixture samples, and the absorption peaks for POM/TBOP-TFSI samples are at 2975, 2917, and 2788 cm^−1^, respectively, indicating the electrostatic interaction between TBOP-TFSI and POM. Because of the existence of this interaction, POM and TBOP-TFSI are partially miscible, consisting of the morphology of composites (as shown in Figure 1).

### 3.2. Crystallization Behaviors of POM/TBOP-TFSI Blends

**Figure 3:**
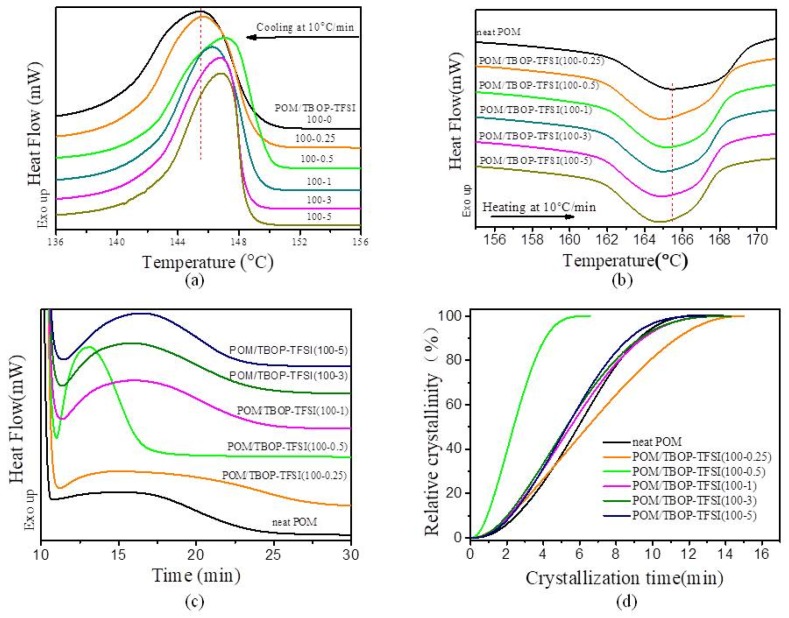
Differential scanning calorimetry (DSC) curves for POM/TBOP-TFSI composites: (**a**) first cooling and (**b**) second heating (after 5 min isothermal for erasing heat history); (**c**) isothermal crystallization process at 152 °C; (**d**) relative crystallinity as a function of crystallization time at 152 °C.

The effects of TBOP-TFSI on the crystallization of POM have been investigated by isothermal and non-isothermal crystallization. It was found that TBOP-TFSI improves both the crystallization temperature and degree of crystallinity of POM as Table 1 shows, indicating that the incorporation of TBOP-TFSI accelerates the crystallization of POM. The crystallinity of POM in all samples was determined by Equation (1).

(1) $$\chi_{c}=\Delta H_{m}/(W_{m}\times \Delta H_{m}^{\circ })\times 100\%$$

where $\Delta H_{m}^{\circ }$ is the theoretical melting enthalpy of POM with a value of 247 J/g [9] and W_m_ is the weight fraction of matrix in composites. Figure 3c,d reveal that the isothermal crystallization time for POM blending with TBOP-TFSI is shorter than that of neat POM. For the POM blended with 0.5 wt% TBOP-TFSI, the t_1/2_ is about 2.4 min, while it takes neat POM for about 5.9 min at 152 °C. The isothermal crystallization behaviors of POM/TBOP-TFSI blends are consistent with the non-isothermal crystallization behaviors, as shown in Figure 3a,b. What is more, the value of (T_m_–T_c_) for POM becomes lower, which means the incorporation of TBOP-TFSI results in a fine crystal grain. From both isothermal and non-isothermal crystallization of POM/TBOP-TFSI thin films, 0.5 wt% addition of ionic liquid, showed a better nucleating ability than other content. We believed this was due to homegeneous dispersion of ionic liquid within the matrix as they were compatible with less than 0.5 wt% content, and also responsible for enhanced mechanical performance.

**Figure 4:**
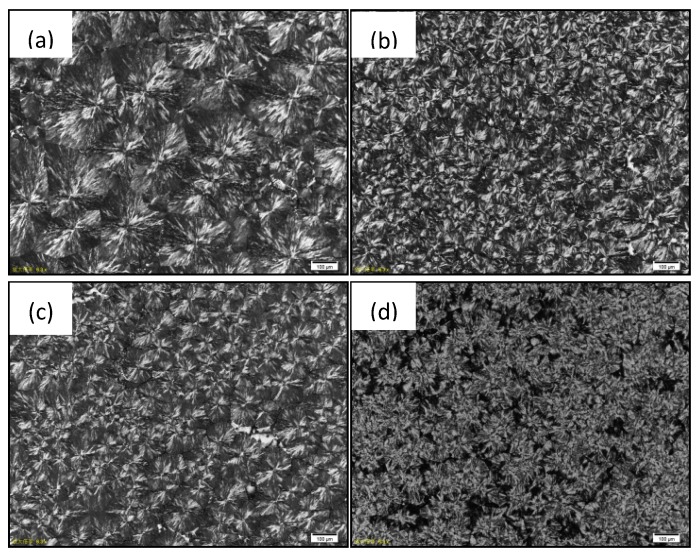
Polarized light microscopy (PLM) images of (**a**) neat POM; (**b**) POM/TBOP-TFSI (100-0.5); (**c**) POM/TBOP-TFSI (100-1); (**d**) POM/TBOP-TFSI (100-3) isothermally crystallized at 152 °C.

The nucleation effect of the ionic liquid can also be confirmed by optical polarize microscopy as in Figure 4. The sizes of POM spherulites became smaller with more loading of TBOP-TFSI (370 μm of neat POM spherulites decreased to 98 μm with 3 wt% of TBOP-TFSI loading). With higher crystal nucleation density and fine crystal grain, the mechanical properties of POM blends achieved a satisfactory enhancement.

### 3.3. Mechanical Properties of POM/TBOP-TFSI Blends

**Figure 5:**
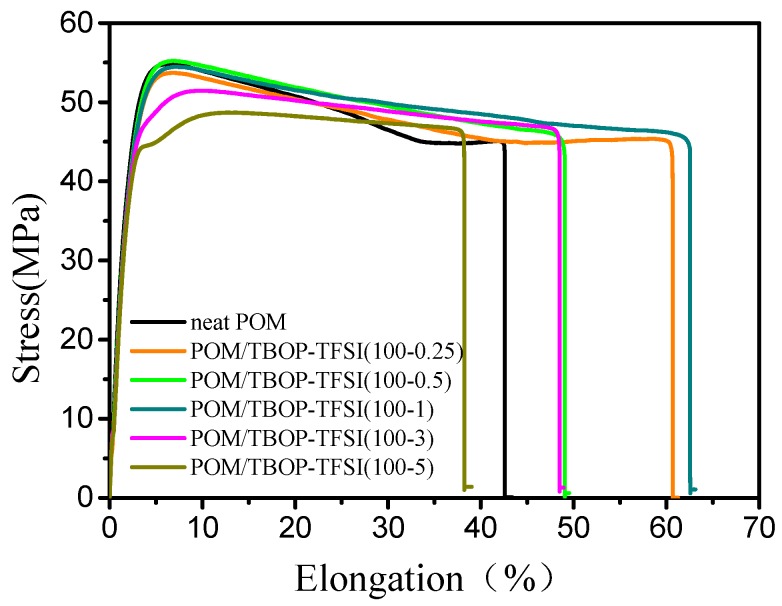
Stress-elongation curves of POM/TBOP-TFSI composites.

The strain–stress curves of POM and POM/TBOP-TFSI specimens are shown in Figure 5 (Table 2). One can observe a significant enhancement of elongation at the break of POM with the addition of a small amount of TBOP-TFSI. At the same time, the tensile strength has a slight improvement. Furthermore, the impact strength of the blends also increases to 4.7 kJ/m^2^ with 0.5 wt% TBOP-TFSI loading as compared with neat POM (4.25 kJ/m^2^), as shown in the exact values of mechanical properties listed in Table 2. In general, the addition of a small amount TBOP-TFSI not only improves the ductility but also maintains the strength of POM matrix. We believe that the smaller sizes of spherulites induced by ionic liquid nucleation were responsible for improved mechanical performance, especially impact strength. However, the addition of more ionic liquid induced worse mechanical properties due to phase separation between TBOP-TFSI and matrix as showed in Figure 2.

### 3.4. Antistatic Properties of POM/TBOP-TFSI Blends

**Figure 6:**
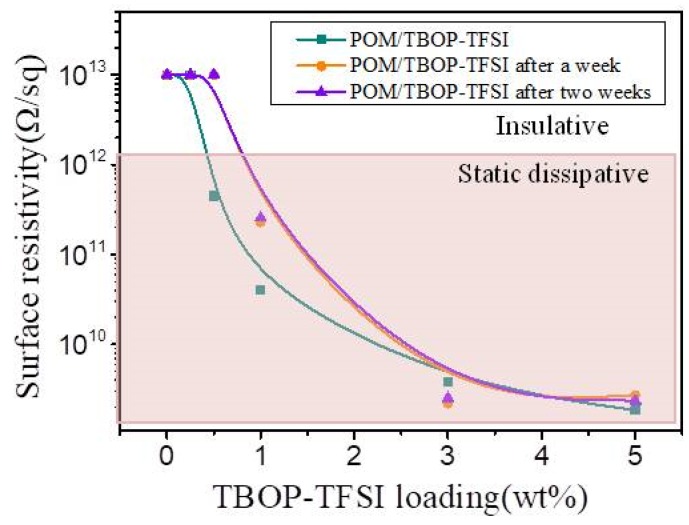
Surface resistivity of POM/TBOP-TFSI composites.

The combination of ionic liquid usually results in the antistatic property for polymer blends. Figure 6 shows the electrical conductivity of POM/TBOP-TFSI films corresponding to TBOP-TFSI loading. It is illustrated that the electrical conductivity of POM/TBOP-TFSI blends decreases with the increasing of TBOP-TFSI content. Generally, the surface resistivity of antistatic material is less than 10^12^ Ω/sq, and neat POM is an insulating material with a surface resistivity higher than 10^13^ Ω/sq. It could be observed in Figure 6 that trace amounts of ionic liquid would be adequate for fabricating antistatic POM materials. The antistatic property can be achieved with only 0.5 wt% TBOP-TFSI addition which indicates the formation of a continuous conducting network within the matrix. The surface resistivity could be further reduced by increasing TBOP-TFSI loading. For blends containing 3 wt% of TBOP-TFSI, the surface resistivity is 3.78 × 10^9^ Ω/sq, which was 2 orders of magnitude less than the 0.5 wt% one. Although electrical conductivity of POM blends could be improved by higher addition of TBOP-TFSI, an excess ionic liquid bleeding was observed for blends with more than 3 wt% TBOP-TFSI loading during mixing for their moderate compatibility with POM, which is unfavorable for mechanical property. Moreover, the values of surface resistivity of all blends stayed almost the same or even lower after storage for a few weeks. The stable antistatic property resulted from partial compatibility of TBOP-TFSI and POM matrix (Figure 1). Ionic liquids were found miscible with POM at very low content (0.5 wt%), leading to the formation of a conductive path due to homogeneous dispersion of TBOP-TFSI in the matrix. At relatively high loadings (1 wt%), although TBOP-TFSI started to aggregate, there were still no large domains observed. One can still obtain the reduced surface resistivity. This excellent long term antistatic performance is of crucial importance for practical applications as internal dust-free parts in electronic devices and industrial structural components that meet the requirement of both mechanical strength and dissipation of static electricity. 

## 4. Conclusions

We investigated crystallization behavior of POM with a very small amount of ionic liquid in binary blends. In POM/TBOP-TFSI blends, ionic liquid acted as a nucleation agent for POM molecules, leading to a higher crystallization temperature and a shorter crystallization time. The size of POM spherulites decreased magnificently as nucleation density increased with ionic liquid addition. Due to the nucleation effect, enhanced mechanical properties could be achieved for POM/TBOP-TFSI binary blends. Furthermore, binary blends exhibited stable surface conductivity which might extend the practical application of POM.

## Figures and Tables

**Table 1:** Differential scanning calorimetry (DSC) results of polyoxymethylene (POM)/tributyl(octyl)phosphonium bis(trifloromethanesulfonyl) imide (TBOP-TFSI) composites.

Samples	T_c_ (°C)	T_m_ (°C)	T_m_–T_c_ (°C)	t_1/2_ (min) at 152 °C	χ_c_ (%)
Neat POM	145.5	165.4	19.9	5.9	51.1
POM/TBOP-TFSI (100-0.25)	145.7	164.9	19.2	6.4	51.3
POM/TBOP-TFSI (100-0.5)	147.1	165.2	18.1	2.4	53.2
POM/TBOP-TFSI (100-1)	146.2	165.0	18.8	5.5	53.6
POM/TBOP-TFSI (100-3)	146.8	164.9	18.1	5.3	52.9
POM/TBOP-TFSI (100-5)	146.8	164.8	18.0	5.3	52.0

**Table 2:** Mechanical properties of POM/TBOP-TFSI composites.

Sample	Yeilding Strength (MPa)	Elongation at Break (%)	Impact Strength (kJ/m^2^)
Neat POM	55.2 ± 0.8	40.2 ± 10	4.2
POM/TBOP-TFSI (100-0.25)	53.8 ± 0.7	56.1 ± 11	4.6
POM/TBOP-TFSI (100-0.5)	54.7 ± 0.5	47.0 ± 9	4.7
POM/TBOP-TFSI (100-1)	54.2 ± 0.8	63.3 ± 12	4.5
POM/TBOP-TFSI (100-3)	51.5 ± 0.5	42.0 ± 6	4.4
POM/TBOP-TFSI (100-5)	48.3 ± 1.0	39.2 ± 13	4.5
